# The genome sequence of a Tiger Cranefly, *Nephrotoma flavescens* (Linnaeus, 1758)

**DOI:** 10.12688/wellcomeopenres.19203.1

**Published:** 2023-03-31

**Authors:** Olga Sivell, Duncan Sivell, Natural History Museum Genome Acquisition Lab, Darwin Tree of Life Barcoding collective, Wellcome Sanger Institute Tree of Life programme, Wellcome Sanger Institute Scientific Operations: DNA Pipelines collective, Tree of Life Core Informatics collective, Darwin Tree of Life Consortium

**Affiliations:** 1 Natural History Museum, London, England, UK

**Keywords:** Nephrotoma flavescens, Tiger Cranefly, genome sequence, chromosomal, Diptera

## Abstract

We present a genome assembly from an individual male *Nephrotoma flavescens* (a Tiger Cranefly; Arthropoda; Insecta; Diptera; Tipulidae). The genome sequence is 1,051.3 megabases in span. Most of the assembly is scaffolded into four chromosomal pseudomolecules, including a partial X sex chromosome. The mitochondrial genome has also been assembled and is 18.9 kilobases in length. Gene annotation of this assembly on Ensembl identified 11,276 protein coding genes.

## Species taxonomy

Eukaryota; Metazoa; Ecdysozoa; Arthropoda; Hexapoda; Insecta; Pterygota; Neoptera; Endopterygota; Diptera; Nematocera; Tipuloidea; Tipulidae; *Nephrotoma*; *Nephrotoma flavescens* (Linnaeus, 1758) (NCBI:txid2719080).

## Background

*Nephrotoma flavescens* is a large elongate fly (wing length 11–14 mm) with long thin legs. It belongs to the family Tipulidae (Diptera) commonly called craneflies. Species from the genus *Nephrotoma* are yellow and black and are often referred to as Tiger craneflies. *Nephrotoma flavescens* can be distinguished from other *Nephrotoma* species by its pale brown stigma, black narrow dorsal stripe on the abdomen, broad black patch on the back of the head and shining black stripe on the prescutum ( Stubbs, 2021).

The species is common in eastern England, but is less common and has a mainly coastal distribution in western England and Wales. In Scotland, *N. flavescens* is widespread in the south and east, but is uncommon in the west Highlands. It prefers dry conditions and can be found on calcareous grassland, dry neutral grassland, richer types of sandy heath or grassland, and also in rough verges or field edges. The adults can be found from June to July or August, rarely in September ( Stubbs, 2021).

The female oviposits into the soil. In laboratory conditions in Lithuania, the eggs hatch after 23 days at 18°C to 25°C ( Podeniene *et al.*, 2014). The larvae, commonly called leatherjackets, are greyish-brown. They feed on plants, mainly the underground parts ( Colyer & Hammond, 1968), potentially causing commercial losses ( Colyer & Hammond, 1968; Hofsvang, 2010), although their economic impact may be questionable, as according to Stubbs (2021) this species avoids improved soils. The last instar larvae have been described by Podeniene (2003) and the first instar larvae by Podeniene *et al.* (2014).

The genome of *Nephrotoma flavescens* was sequenced as part of the Darwin Tree of Life Project, a collaborative effort to sequence all named eukaryotic species in the Atlantic Archipelago of Britain and Ireland. Here we present a chromosomally complete genome sequence based on one male specimen from an urban garden in Luton. This genome note will aid research on the phylogeny, taxonomy, biology and ecology of the species.

### Genome sequence report

**Figure 1.**
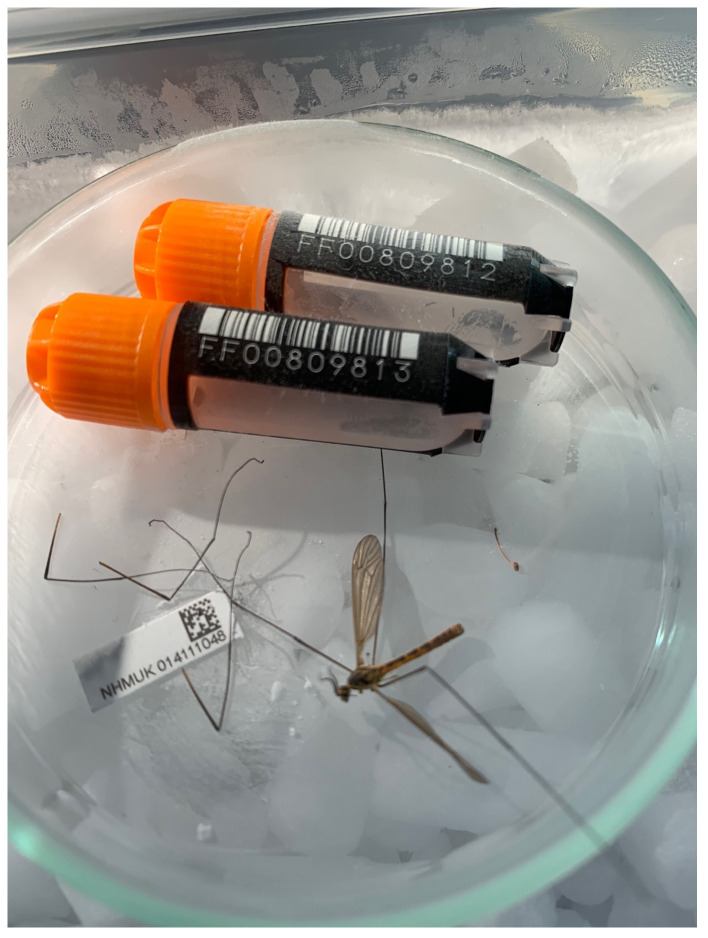
Photograph of the *Nephrotoma flavescens* (idNepFlae1) specimen used for genome sequencing.

The genome was sequenced from one male *Nephrotoma flavescens* specimen ( Figure 1) collected from Luton, UK (latitude 51.89, longitude –0.39). A total of 35-fold coverage in Pacific Biosciences single-molecule HiFi long reads was generated. Primary assembly contigs were scaffolded with chromosome conformation Hi-C data. Manual assembly curation corrected 214 missing or mis-joins and removed eight haplotypic duplications, reducing the assembly length by 0.26% and the scaffold number by 7.7%, and increasing the scaffold N50 by 1.35%.

**Figure 2.**
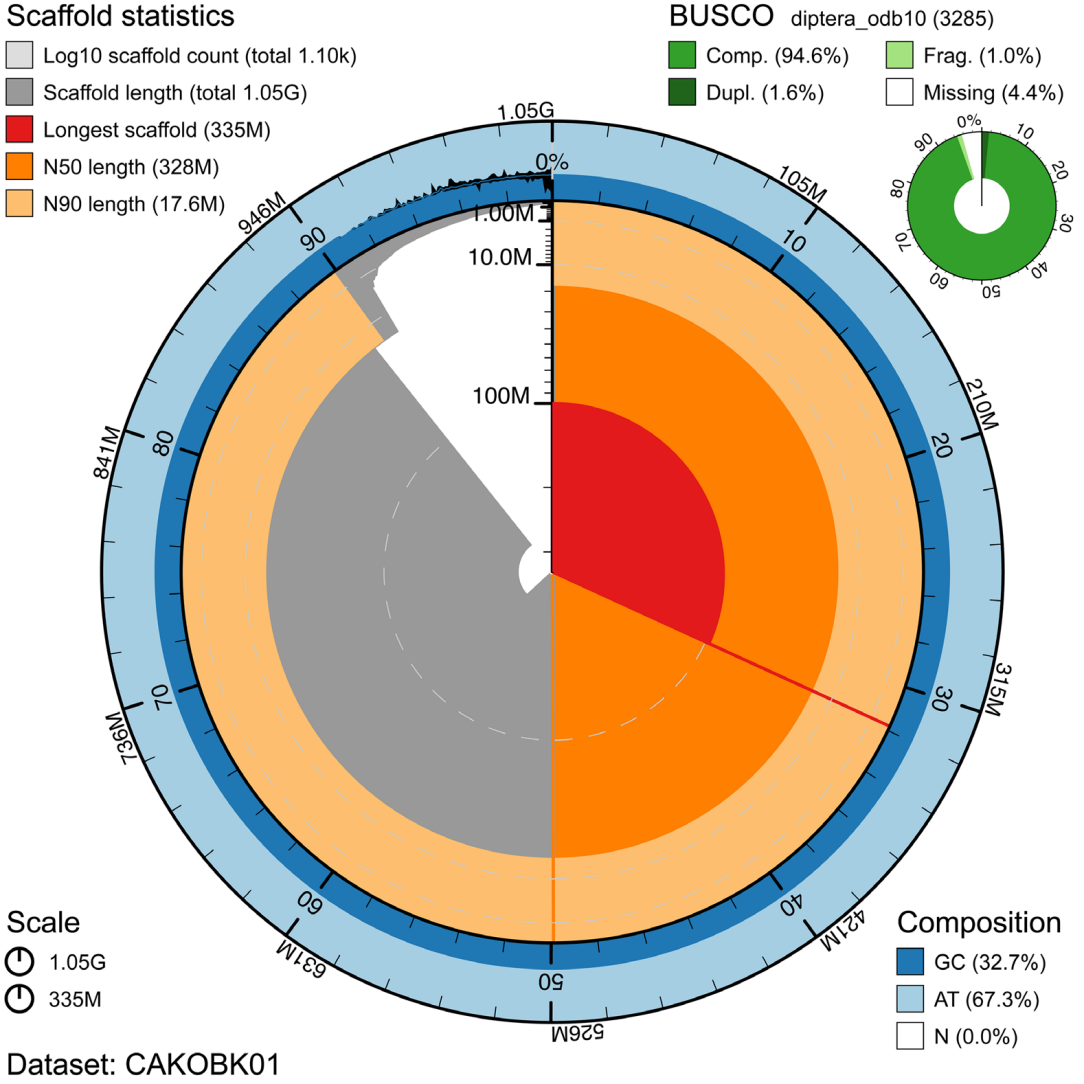
Genome assembly of *Nephrotoma flavescens*, idNepFlae1.1: metrics. The BlobToolKit Snailplot shows N50 metrics and BUSCO gene completeness. The main plot is divided into 1,000 size-ordered bins around the circumference with each bin representing 0.1% of the 1,051,351,689 bp assembly. The distribution of scaffold lengths is shown in dark grey with the plot radius scaled to the longest scaffold present in the assembly (334,972,678 bp, shown in red). Orange and pale-orange arcs show the N50 and N90 scaffold lengths (327,956,322 and 17,607,469 bp), respectively. The pale grey spiral shows the cumulative scaffold count on a log scale with white scale lines showing successive orders of magnitude. The blue and pale-blue area around the outside of the plot shows the distribution of GC, AT and N percentages in the same bins as the inner plot. A summary of complete, fragmented, duplicated and missing BUSCO genes in the diptera_odb10 set is shown in the top right. An interactive version of this figure is available at https://blobtoolkit.genomehubs.org/view/idNepFlae1.1/dataset/CAKOBK01/snail.

The final assembly has a total length of 1,051.3 Mb in 1103 sequence scaffolds with a scaffold N50 of 328.0 Mb ( Table 1). Most (90.97%) of the assembly sequence was assigned to four chromosomal-level scaffolds, representing three autosomes, and a partial X sex chromosome. Chromosome-scale scaffolds confirmed by the Hi-C data are named in order of size ( Figure 2– Figure 5; Table 2). It is likely that there is a Y chromosome, and that X and Y are highly repetitive, fragmented and for the most part indistinguishable.

**Table 1.** Genome data for *Nephrotoma flavescens*, idNepFlae1.1.

Project accession data		
Assembly identifier	idNepFlae1.1	
Species	*Nephrotoma flavescens*	
Specimen	idNepFlae1	
NCBI taxonomy ID	2719080	
BioProject	PRJEB50880	
BioSample ID	SAMEA7520954	
Isolate information	idNepFlae1 (PacBio); idNepFlae2 (RNA-Seq and Hi-C)	
Assembly metrics *		*Benchmark*
Consensus quality (QV)	56.8	*≥ 50*
*k*-mer completeness	99.99%	*≥ 95%*
BUSCO **	C:94.6%[S:93.0%,D:1.6%], F:1.0%,M:4.4%,n:3,285	*C ≥ 95%*
Percentage of assembly mapped to chromosomes	90.97%	*≥ 95%*
Sex chromosomes	Partial X chromosome	*localised homologous pairs*
Organelles	Mitochondrial genome assembled	*complete single alleles*
Raw data accessions		
PacificBiosciences SEQUEL II	ERR8705863, ERR8705864	
Hi-C Illumina	ERR8702788	
PolyA RNA-Seq Illumina	ERR10123678	
Genome assembly		
Assembly accession	GCA_932526605.1	
*Accession of alternate haplotype*	GCA_932525705.1	
Span (Mb)	1,051.3	
Number of contigs	2,049	
Contig N50 length (Mb)	2.9	
Number of scaffolds	1,103	
Scaffold N50 length (Mb)	328.0	
Longest scaffold (Mb)	335.0	
Genome annotation		
Number of protein-coding genes	11,276	
Number of non-coding genes	2,990	
Number of gene transcripts	19,886	

* Assembly metric benchmarks are adapted from column VGP-2020 of “Table 1: Proposed standards and metrics for defining genome assembly quality” from ( Rhie *et al.*, 2021). ** BUSCO scores based on the diptera_odb10 BUSCO set using v5.3.2. C = complete [S = single copy, D = duplicated], F = fragmented, M = missing, n = number of orthologues in comparison. A full set of BUSCO scores is available at https://blobtoolkit.genomehubs.org/view/idNepFlae1.1/dataset/CAKOBK01/busco.

**Figure 3.**
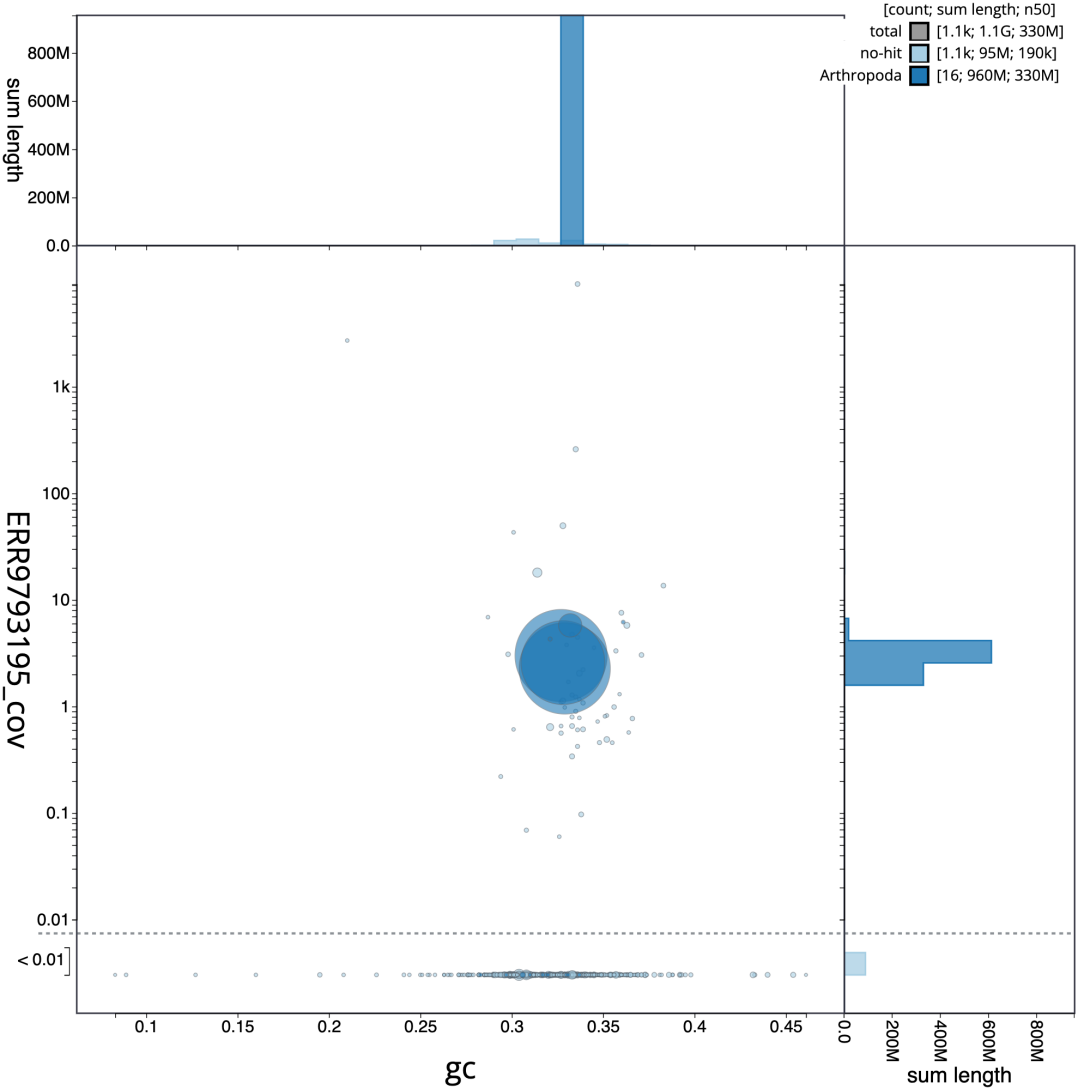
Genome assembly of *Nephrotoma flavescens*, idNepFlae1.1: GC coverage. BlobToolKit GC-coverage plot. Scaffolds are coloured by phylum. Circles are sized in proportion to scaffold length. Histograms show the distribution of scaffold length sum along each axis. An interactive version of this figure is available at https://blobtoolkit.genomehubs.org/view/idNepFlae1.1/dataset/CAKOBK01/blob.

**Figure 4.**
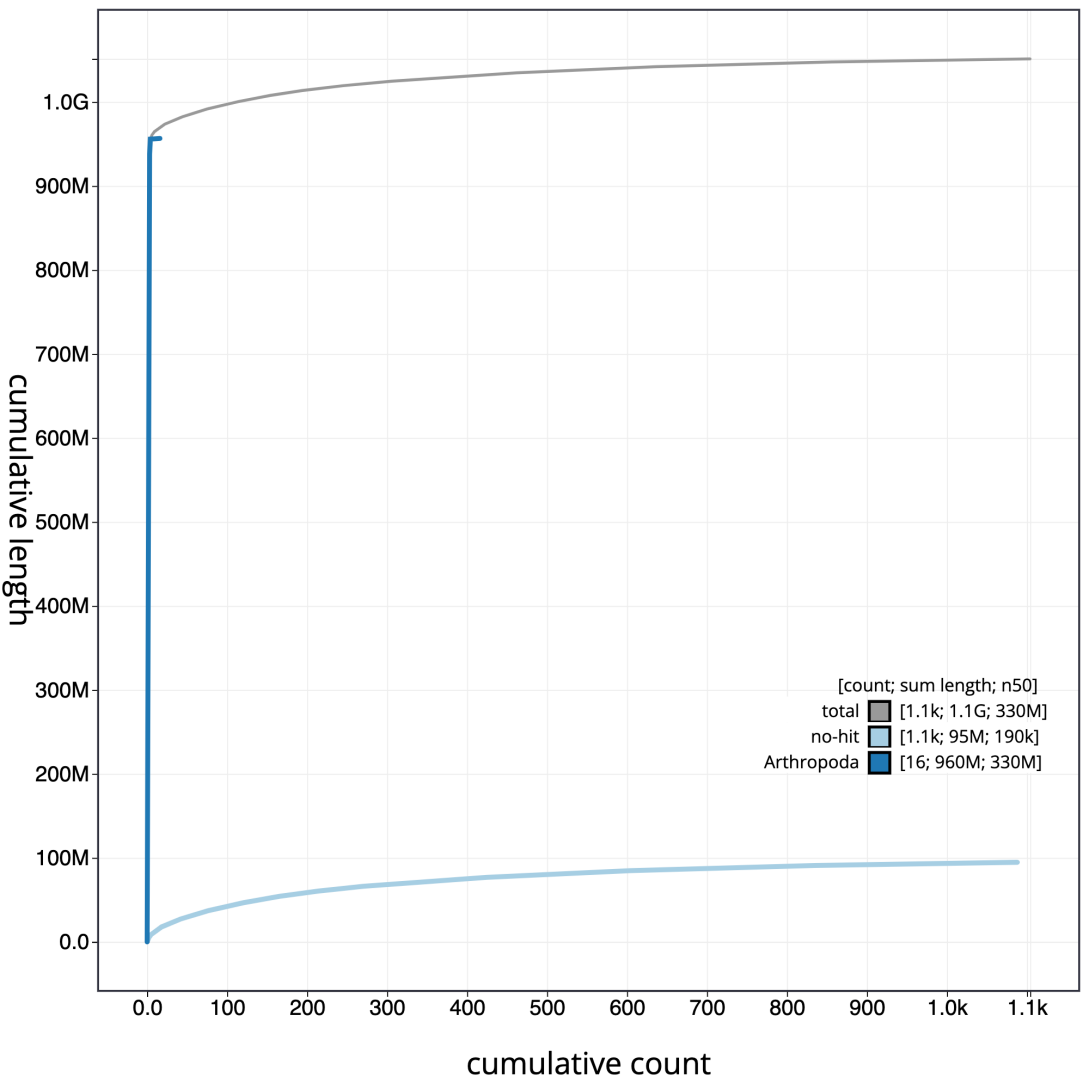
Genome assembly of *Nephrotoma flavescens*, idNepFlae1.1: cumulative sequence. BlobToolKit cumulative sequence plot. The grey line shows cumulative length for all scaffolds. Coloured lines show cumulative lengths of scaffolds assigned to each phylum using the buscogenes taxrule. An interactive version of this figure is available at https://blobtoolkit.genomehubs.org/view/idNepFlae1.1/dataset/CAKOBK01/cumulative.

**Figure 5.**
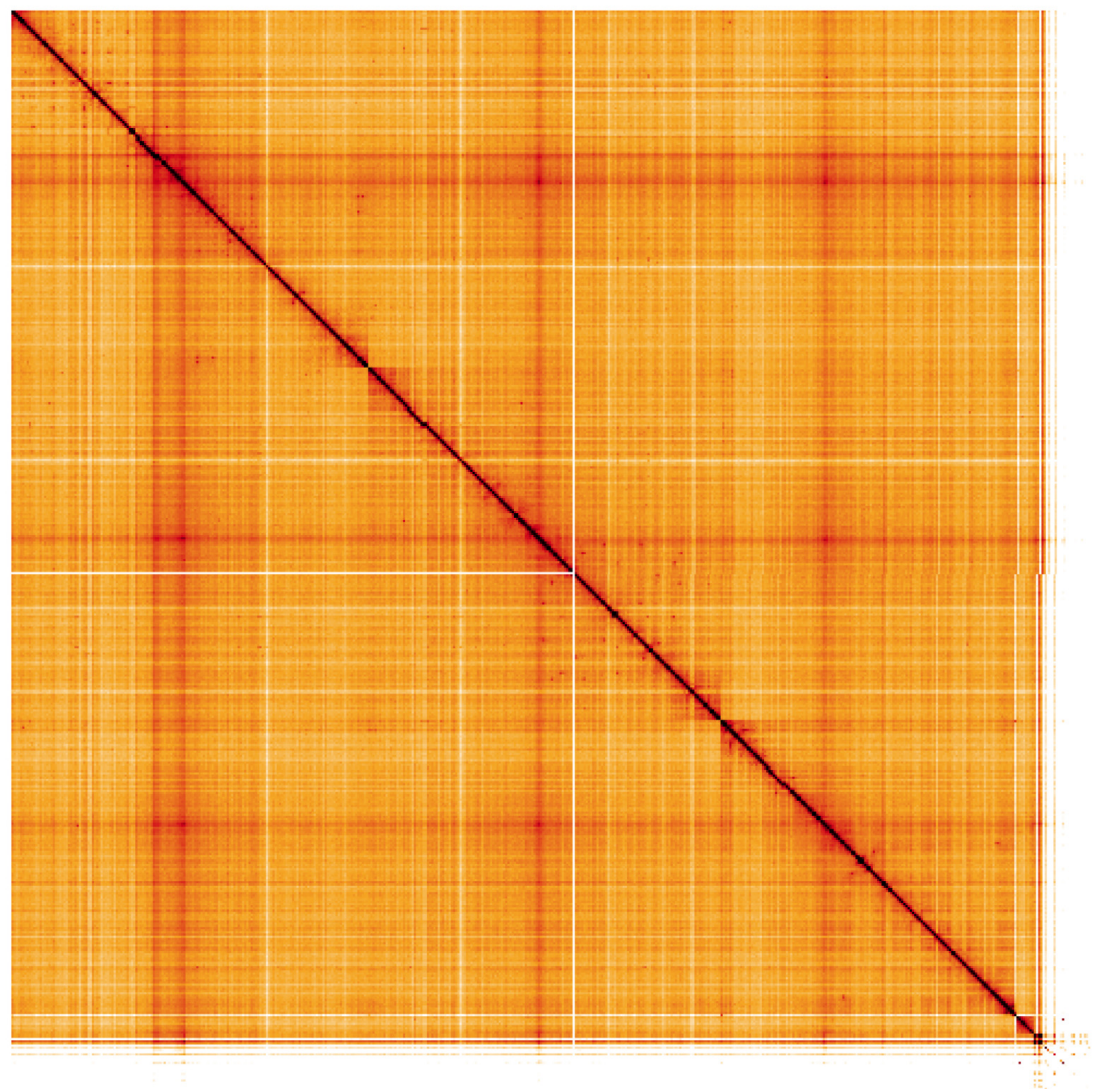
Genome assembly of *Nephrotoma flavescens*, idNepFlae1.1: Hi-C contact map. Hi-C contact map of the idNepFlae1.1 assembly, visualised using HiGlass. Chromosomes are shown in order of size from left to right and top to bottom. An interactive version of this figure may be viewed at https://genome-note-higlass.tol.sanger.ac.uk/l/?d=J47DPOhJSyayTcYU59Gjyg.

**Table 2.** Chromosomal pseudomolecules in the genome assembly of *Nephrotoma flavescens*, idNepFlae1.

INSDC accession	Chromosome	Size (Mb)	GC%
OW052220.1	1	334.97	32.7
OW052221.1	2	327.96	32.9
OW052222.1	3	275.27	32.8
OW052223.1	X	17.61	33.2
OW052224.1	MT	0.02	21
-	unplaced	95.53	31.9

The assembly has a BUSCO v5.3.2 ( Manni *et al.*, 2021) completeness of 94.6% (single 93.0%, duplicated 1.6%) using the diptera_odb10 reference set. While not fully phased, the assembly deposited is of one haplotype. Contigs corresponding to the second haplotype have also been deposited.

### Genome annotation report

The *Nephrotoma flavescens* (idNepFlae1.1) genome assembly was annotated using the Ensembl rapid annotation pipeline ( Table 1; Ensembl Accession number GCA_932526605.1). The resulting annotation includes 19,886 transcribed mRNAs from 11,276 protein-coding and 2,990 non-coding genes.

## Methods

### Sample acquisition and nucleic acid extraction

One male *Nephrotoma flavescens* specimen ( Figure 1) was collected by Olga Sivell (Natural History Museum, London) on 2 June 2020 by netting in a private urban garden in Luton (51.89, –0.39). This specimen (NHMUK014111048; idNepFlae1) was used for genome sequencing. Another *N. flavescens* specimen was collected from the same place on 16 June 2020. This specimen (NHMUK014111058; idNepFlae2) was used for RNA sequencing and Hi-C scaffolding. Both specimens were identified by Duncan Sivell (Natural History Museum, London) using ( Boardman, 2016) and test keys that have since been published in Stubbs (2021). The specimens were preserved on dry ice.

DNA was extracted at the Tree of Life laboratory, Wellcome Sanger Institute (WSI). The idNepFlae1 sample was weighed and dissected on dry ice. Head and thorax tissue was disrupted using a Nippi Powermasher fitted with a BioMasher pestle. High molecular weight (HMW) DNA was extracted using the Qiagen MagAttract HMW DNA extraction kit. HMW DNA was sheared into an average fragment size of 12–20 kb in a Megaruptor 3 system with speed setting 30. Sheared DNA was purified by solid-phase reversible immobilisation using AMPure PB beads with a 1.8X ratio of beads to sample to remove the shorter fragments and concentrate the DNA sample. The concentration of the sheared and purified DNA was assessed using a Nanodrop spectrophotometer and Qubit Fluorometer and Qubit dsDNA High Sensitivity Assay kit. Fragment size distribution was evaluated by running the sample on the FemtoPulse system.

RNA was extracted from abdomen tissue of idNepFlae2 in the Tree of Life Laboratory at the WSI using TRIzol, according to the manufacturer’s instructions. RNA was then eluted in 50 μL RNAse-free water and its concentration assessed using a Nanodrop spectrophotometer and Qubit Fluorometer using the Qubit RNA Broad-Range (BR) Assay kit. Analysis of the integrity of the RNA was done using Agilent RNA 6000 Pico Kit and Eukaryotic Total RNA assay.

### Sequencing

Pacific Biosciences HiFi circular consensus DNA sequencing libraries were constructed according to the manufacturers’ instructions. Poly(A) RNA-Seq libraries were constructed using the NEB Ultra II RNA Library Prep kit. DNA and RNA sequencing was performed by the Scientific Operations core at the WSI on Pacific Biosciences SEQUEL II (HiFi) and Illumina NovaSeq 6000 (RNA-Seq). Hi-C data were also generated from head tissue of idNepFlae2 using the Arima v2 kit and sequenced on the Illumina NovaSeq 6000 instrument.

### Genome assembly

Assembly was carried out with Hifiasm ( Cheng *et al.*, 2021) and haplotypic duplication was identified and removed with purge_dups ( Guan *et al.*, 2020). The assembly was scaffolded with Hi-C data ( Rao *et al.*, 2014) using YaHS ( Zhou *et al.*, 2023). The assembly was checked for contamination as described previously ( Howe *et al.*, 2021). Manual curation was performed using HiGlass ( Kerpedjiev *et al.*, 2018) and Pretext ( Harry, 2022). The mitochondrial genome was assembled using MitoHiFi ( Uliano-Silva *et al.*, 2022), which performed annotation using MitoFinder ( Allio *et al.*, 2020). The genome was analysed, and BUSCO scores were generated within the BlobToolKit environment ( Challis *et al.*, 2020). Table 3 contains a list of all software tool versions used, where appropriate.

**Table 3.** Software tools and versions used.

Software tool	Version	Source
BlobToolKit	3.4.0	Challis *et al.*, 2020
Hifiasm	0.16.1-r375	Cheng *et al.*, 2021
HiGlass	1.11.6	Kerpedjiev *et al.*, 2018
MitoHiFi	2	Uliano-Silva *et al.*, 2022
PretextView	0.2	Harry, 2022
purge_dups	1.2.3	Guan *et al.*, 2020
YaHS	yahs-1.1.91eebc2	Zhou *et al.*, 2023

### Genome annotation

The Ensembl gene annotation system ( Aken *et al.*, 2016) was used to generate annotation for the *Nephrotoma flavescens* assembly (GCA_932526605.1). Annotation was created primarily through alignment of transcriptomic data to the genome, with gap filling via protein-to-genome alignments of a select set of proteins from UniProt ( UniProt Consortium, 2019).

### Ethics and compliance issues

The materials that have contributed to this genome note have been supplied by a Darwin Tree of Life Partner. The submission of materials by a Darwin Tree of Life Partner is subject to the Darwin Tree of Life Project Sampling Code of Practice. By agreeing with and signing up to the Sampling Code of Practice, the Darwin Tree of Life Partner agrees they will meet the legal and ethical requirements and standards set out within this document in respect of all samples acquired for, and supplied to, the Darwin Tree of Life Project. All efforts are undertaken to minimise the suffering of animals used for sequencing. Each transfer of samples is further undertaken according to a Research Collaboration Agreement or Material Transfer Agreement entered into by the Darwin Tree of Life Partner, Genome Research Limited (operating as the Wellcome Sanger Institute), and in some circumstances other Darwin Tree of Life collaborators.

## Data Availability

European Nucleotide Archive: *Nephrotoma flavescens* (cranefly). Accession number PRJEB50880; https://identifiers.org/ena.embl/PRJEB50880 ( Wellcome Sanger Institute, 2022) The genome sequence is released openly for reuse. The *Nephrotoma flavescens* genome sequencing initiative is part of the Darwin Tree of Life (DToL) project. All raw sequence data and the assembly have been deposited in INSDC databases. Raw data and assembly accession identifiers are reported in Table 1.

## Author information

Members of the Natural History Museum Genome Acquisition Lab are listed here:

https://doi.org/10.5281/zenodo.4790042.

Members of the Darwin Tree of Life Barcoding collective are listed here: https://doi.org/10.5281/zenodo.4893703.

Members of the Wellcome Sanger Institute Tree of Life programme are listed here: https://doi.org/10.5281/zenodo.4783585.

Members of Wellcome Sanger Institute Scientific Operations: DNA Pipelines collective are listed here: https://doi.org/10.5281/zenodo.4790455.

Members of the Tree of Life Core Informatics collective are listed here: https://doi.org/10.5281/zenodo.5013541.

Members of the Darwin Tree of Life Consortium are listed here: https://doi.org/10.5281/zenodo.4783558.

## References

[ref-1] Aken BL Ayling S Barrell D : The Ensembl gene annotation system. *Database (Oxford).* 2016;baw093. 10.1093/database/baw093 27337980 PMC4919035

[ref-2] Allio R Schomaker-Bastos A Romiguier J : MitoFinder: Efficient automated large‐scale extraction of mitogenomic data in target enrichment phylogenomics. *Mol Ecol Resour.* 2020;20(4):892–905. 10.1111/1755-0998.13160 32243090 PMC7497042

[ref-3] Boardman P : Shropshire craneflies.FSC (Field Studies Council) Publications.2016. Reference Source

[ref-4] Challis R Richards E Rajan J : BlobToolKit - interactive quality assessment of genome assemblies. *G3 (Bethesda).* 2020;10(4):1361–1374. 10.1534/g3.119.400908 32071071 PMC7144090

[ref-5] Cheng H Concepcion GT Feng X : Haplotype-resolved *de novo* assembly using phased assembly graphs with hifiasm. *Nat Methods.* 2021;18(2):170–175. 10.1038/s41592-020-01056-5 33526886 PMC7961889

[ref-6] Colyer CN Hammond C : Flies of the British Isles.London: Warne.1968. Reference Source

[ref-7] Guan D McCarthy SA Wood J : Identifying and removing haplotypic duplication in primary genome assemblies. *Bioinformatics.* 2020;36(9):2896–2898. 10.1093/bioinformatics/btaa025 31971576 PMC7203741

[ref-8] Harry E : PretextView (Paired REad TEXTure Viewer): A desktop application for viewing pretext contact maps.2022; (Accessed: 19 October 2022). Reference Source

[ref-9] Hofsvang T : *Nephrotoma* Meigen, 1803 (Diptera, Tipulidae) as potential agricultural pests in Norway. *Nor J Entomol.* 2010;57(1):12–16. Reference Source

[ref-10] Howe K Chow W Collins J : Significantly improving the quality of genome assemblies through curation. *GigaScience.* Oxford University Press.2021;10(1):giaa153. 10.1093/gigascience/giaa153 33420778 PMC7794651

[ref-11] Kerpedjiev P Abdennur N Lekschas F : HiGlass: Web-based visual exploration and analysis of genome interaction maps. *Genome Biol.* 2018;19(1):125. 10.1186/s13059-018-1486-1 30143029 PMC6109259

[ref-12] Manni M Berkeley MR Seppey M : BUSCO Update: Novel and Streamlined Workflows along with Broader and Deeper Phylogenetic Coverage for Scoring of Eukaryotic, Prokaryotic, and Viral Genomes. *Mol Biol Evol.* 2021;38(10):4647–4654. 10.1093/molbev/msab199 34320186 PMC8476166

[ref-13] Podeniene V : Morphology and ecology of the last instar larvae of the crane flies (Diptera, Tipulomorpha) of Lithuania (in Lithuanian).Doctoral dissertation. Vilnius University.2003.

[ref-14] Podeniene V Naseviciene N Podenas S : Notes on the first instar larvae of *Ctenophora* and *Nephrotoma* (Diptera, Tipulidae). *Zootaxa.* 2014;3764(2):152–168. 10.11646/zootaxa.3764.2.3 24870629

[ref-15] Rao SSP Huntley MH Durand NC : A 3D map of the human genome at kilobase resolution reveals principles of chromatin looping. *Cell.* 2014;159(7):1665–1680. 10.1016/j.cell.2014.11.021 25497547 PMC5635824

[ref-16] Rhie A McCarthy SA Fedrigo O : Towards complete and error-free genome assemblies of all vertebrate species. *Nature.* 2021;592(7856):737–746. 10.1038/s41586-021-03451-0 33911273 PMC8081667

[ref-17] Stubbs AE : British craneflies.British Entomological and Natural History Society. 2021. Reference Source

[ref-18] Uliano-Silva M Ferreira JGRN Krasheninnikova K : MitoHiFi: a python pipeline for mitochondrial genome assembly from PacBio High Fidelity reads. *bioRxiv.* [Preprint].2022. 10.1101/2022.12.23.521667 PMC10354987 37464285

[ref-19] UniProt Consortium: UniProt: a worldwide hub of protein knowledge. *Nucleic Acids Res.* 2019;47(D1):D506–D515. 10.1093/nar/gky1049 30395287 PMC6323992

[ref-21] Wellcome Sanger Institute: The genome sequence of a Tiger Cranefly, Nephrotoma flavescens (Linnaeus, 1758). European Nucleotide Archive.[dataset], accession number PRJEB50880,2022.

[ref-20] Zhou C McCarthy SA Durbin R : YaHS: yet another Hi-C scaffolding tool. *Bioinformatics.* Edited by C. Alkan,2023;39(1):btac808. 10.1093/bioinformatics/btac808 36525368 PMC9848053

