# Supplementary material for: The genome sequence of a Tiger Cranefly, Nephrotoma flavescens (Linnaeus, 1758)
Source: Wellcome Open Res. 2023 Mar 31;8:148. [Version 1] doi: 10.12688/wellcomeopenres.19203.1 (PMC10238818; doi:10.12688/wellcomeopenres.19203.1)
Supplement: Peer review history

# Peer review: Reviewer response for version 1

**DOI:** 10.21956/wellcomeopenres.21285.r58641

**Published:** 2023-06-07

**Authors:** Rosario Nicoletti

**Affiliations:** 1 University of Naples Federico II, Portici, Italy

This paper reports on the genome sequencing of a cranefly species, *Nephrotoma flavescens*, based on a specimen collected in southern England, for which a morphological description is provided supporting correct taxonomic identification. The work is part of the commendable Darwin Tree of Life Project; it has been carried out with competence and is adequately detailed in terms of methodology and general procedures, leaning towards a positive advice for indexing.

Are sufficient details of methods and materials provided to allow replication by others?

Yes

Is the rationale for creating the dataset(s) clearly described?

Yes

Are the datasets clearly presented in a useable and accessible format?

Yes

Are the protocols appropriate and is the work technically sound?

Yes

Reviewer Expertise:

Mycology, Fungal Diversity, Bioactive Products, Plant Protection, Biocontrol, Ecological Relationships

I confirm that I have read this submission and believe that I have an appropriate level of expertise to confirm that it is of an acceptable scientific standard.

# Peer review: Reviewer response for version 1

**DOI:** 10.21956/wellcomeopenres.21285.r57048

**Published:** 2023-06-02

**Authors:** Balaji Chattopadhyay

**Affiliations:** 1 Ashoka University, Sonipat, Haryana, India

The paper 'The genome sequence of a Tiger Cranefly, *Nephrotoma flavescens* (Linnaeus, 1758)' by Sivell *et al.*,  is brief and the methods are presented with sufficient details. My only suggestion to the authors will be to discuss their results in a little bit more detail, particularly comparing the quality of their assembly with already available genomes *. *I also feel that the methods can be improved further by adding at least the following information:

- Methodological details for quality control of the sequence data.
- Genome size repeat content and heterozygote estimates should be included.
- Purpose and some details of each program should be added, particularly in regards to their benefit over alternatives. For each program, the usage of critical parameters should be mentioned with a justification.

All this information will greatly help others in replicating the analyses and taking an informed decision while performing their own assembly.

 Overall the protocols are fairly appropriate and the paper is methodologically sound. The data is deposited in a public database. I will recommend minor revisions and adding the requested details in the manuscript for more clarity and further details.

Are sufficient details of methods and materials provided to allow replication by others?

Yes

Is the rationale for creating the dataset(s) clearly described?

Yes

Are the datasets clearly presented in a useable and accessible format?

Yes

Are the protocols appropriate and is the work technically sound?

Yes

Reviewer Expertise:

Evolutionary Biology, Biodiversity genomics, Population genomics, Phylogenomics, Comparative Genomics

I confirm that I have read this submission and believe that I have an appropriate level of expertise to confirm that it is of an acceptable scientific standard.

# Peer review: Reviewer response for version 1

**DOI:** 10.21956/wellcomeopenres.21285.r55987

**Published:** 2023-04-19

**Authors:** Xiao Zhang

**Affiliations:** 1 Shandong Engineering Research Center for Environment-Friendly Agricultural Pest Management, College of Plant Health and Medicine, Qingdao Agricultural University, Qingdao, Shandong, China

In this study, the genome sequence of *Nephrotoma flavescens* (Insecta, Diptera, Tipulidae) is presented, which is 1,051.3 megabases in span. The authors provided specific collection time and location for the materials, which were identified by Duncan Sivell (Natural History Museum, London) based on some literature. The work technically is sound and sufficient details of methods are provided. Their sequencing was performed by the Scientific Operations core at the WSI on Pacific Biosciences SEQUEL II (HiFi) and Illumina NovaSeq 6000 (RNA-Seq). Most of the assembly is scaffolded into four chromosomal pseudomolecules, including a partial X sex chromosome. Gene annotation of this assembly on Ensembl identified 11,276 protein coding genes. The sequencing data and assemblies generated for *N. flavescens* in this study are openly available in European Nucleotide Archive (https://www.ebi.ac.uk/ena/browser/home) under the accession number PRJEB50880.

Are sufficient details of methods and materials provided to allow replication by others?

Yes

Is the rationale for creating the dataset(s) clearly described?

Yes

Are the datasets clearly presented in a useable and accessible format?

Yes

Are the protocols appropriate and is the work technically sound?

Yes

Reviewer Expertise:

Systematics (Taxonomy); Phylogenomics; Evolutionary Biology

I confirm that I have read this submission and believe that I have an appropriate level of expertise to confirm that it is of an acceptable scientific standard.
